# Oxidants and antioxidants: friends or foes?

**DOI:** 10.5455/oams.080612.ed.001

**Published:** 2012-06-08

**Authors:** Sukru Oter, Si Jin, Luca Cucullo, H.J. Damien Dorman

**Affiliations:** 1 Department of Physiology, Gulhane Military Medical Academy, Ankara, Turkey; 2 Department of Pharmacology, Tongji Medical College, Huazhong University of Science and Technology, Wuhan, Hubei, PR China; 3 Department of Pharmaceutical Sciences, Texas Tech University Health Sciences Center, Amarillo, TX, USA; 4 Division of Pharmacognosy, Faculty of Pharmacy, University of Helsinki, Finland

**Keywords:** Oxidants, Antioxidants, Free radicals, Reactive species, Redox signalling

Albeit the existence of ‘free radicals’ having been known for a considerable time within the sphere of chemistry, these interesting kinds of oxidizing molecules attracted the attention of medical scientists and physicians during the early 1950s when Denham Harman started to publish a number of reports on the “free radical theory of aging” [1]. Two decades later, grounded in the recognition that free radical production in the cell occurs mainly in the mitochondria and that mutations of the mitochondrial DNA (mtDNA) are strongly involved in the aging process, this theory evolved into the “mitochondrial theory of aging” [2, 3]. Despite the ‘theory’ label, the entire medical world believes, at least in part, in the truth of this explanation for the underlying mechanisms of - the unavoidable biological process - aging.

Starting with the 1970s, overwhelming research began to appear in the medical literature elucidating the relationship between free radicals with this or that pathophysiological condition which resulted in the formulation of the definition of “free radical diseases” [4]. Through this, many pathologies such as essential hypertension, atherosclerosis, autoimmune diseases and cancer were explained by the involvement of free radicals [5]. Depending on the molecular source or basis, the simple term ‘free radical’ became widened by other descriptions such as ‘reactive oxygen species’ or ‘oxygen free radicals’. Short after the discovery by Ignarro et al [6] that the endogenous vascular dilating mediator widely known as the endothelium-derived relaxing factor (EDRF) was nitric oxide (NO^•^), a gaseous radical molecule, another term, namely ‘reactive nitrogen species’ was included to the nomenclature of this particular field of science.

A significant number of radicals such as the superoxide free radical anion (O_2_^•−^) or the hydroxyl radical (^•^OH) and another group of ‘non-radical reactive molecules’ such as hydrogen peroxide (H_2_O_2_) and peroxynitrite (ONOO^−^) were defined and various deleterious effects of these molecules have been described through the past decades [7, 8]. Ultimately, damage to cells by these highly reactive oxygen and nitrogen species (ROS and RNS) occurs as a result of alterations of macromolecules [9, 10]. These include lipoperoxidation of polyunsaturated fatty acids in membrane lipids, protein oxidation, DNA strand breakage [11–14], RNA oxidation [15], mitochondrial depolarization and apoptosis. Mutations of the nuclear protein p53 which may lead to apoptosis are also associated with oxidative stress. Impairments of cellular/tissue functions caused by oxidative stress have been implicated in disease states, *viz.*, Alzheimer’s [16] and Parkinson’s disease [17], various cancers [18], and aging processes [19], amoungst others. Under normal conditions, reactive species are cleared by antioxidants which, broadly speaking, refer to molecules that are able to react directly with oxidants to reduce their oxidation capacity, *e.g.* scavenging enzymes such as superoxide dismutase, catalase, glutathione peroxidase, *etc.*, or chemicals inhibiting the activities of oxidant generating enzymes such as xanthine oxidase, *e.g.* polyphenols. These molecules can be either natural or synthetic, either hydrophilic such as ascorbic acid or hydrophobic such as α-tocopherol. By these actions, antioxidants can either prevent the generation of oxidizing species or reduce the effects of dangerous metabolic or xenobiotic oxidants and hence prevent the body from acute or chronic diseases and/or repair the cellular/tissue damage already sustained. Therefore, it is not surprising that a large number of studies have been concentrated on molecules with antioxidant activity for therapeutic purposes to counteract the harmful effects of reactive species and oxidative stress. It should be noted, however, that a considerable number of antioxidant molecules were instead proved to have pro-oxidant potential and to promote oxidative reactions [20]. Thus, the use of antioxidants for preventing against possible radical-caused injuries, namely “antioxidant therapy”, is today still a controversial issue [21, 22] and may explain often contradictory finding in human trials.

On the other hand, it also became obvious that free radicals are not only involved in pathological processes, but their existence is also necessary for many physiological functions of living organisms [23, 24], including ‘healthy aging’ [25, 26]. Lipid peroxidation, a major consequence of free radical-dependent injury, was also reported to have potential for both deleterious and beneficial effects [27, 28]. It is now widely known that these biologically ‘hyper’-active molecules are acting as signaling agents in various cellular pathways opening a new research era, the so-called “redox signaling” [29–31]. Hydrogen peroxide and peroxynitrite, in particular, have been implicated in a considerable number of cellular signaling cascades [32–34]; depending on their non-radical structure these molecules have a relative longer half-life than almost all other oxidants allowing them to migrate away from their production sites and to diffuse through membranes. Herewith, transcription factors such as AP-1, NF-κB and/or Nrf2 have been reported to be involved in these redox-modulated signaling pathways [35–37].

Taken together, the current concensus is that a controlled and sustained production of both radical and non-radical reactive molecules is essential for normal physiological and cellular functions; however, their uncontrolled or excessive production can cause ‘oxidative/nitrosative stress’ resulting in the destruction of structural biomolecules consequently leading cellular dysfunction and death and ultimately to tissue and organ injury or failure. The scientific world is encouraging engaged in investigating whether oxidants or antioxidants are friends or foes for each other and/or for living organisms; more and more research is being performed in order to clarify the mechanisms of action of endogenously produced oxidizing molecules, their relation to physiological processes and interactions with other biomolecules.

With this first issue of “***Oxidants and Antioxidants in Medical Science***”, we announce a new periodical resource for research professionals in this attractive area in order to find chance to share their experiences and knowledge with medical professionals through the medical literature. We are starting with a 10-article issue from authors and research groups all over the world including the United States, Cuba, Belgium, Estonia, India and Malaysia. The first issue includes both review articles authorized by senior academicians and research papers of the field of redox science. Our main aim is to provide relevant and reliable knowledge for scientists of this field and, by this way, to open a new door to the world and secrets of oxidants and antioxidants in medical science.

## Figures and Tables

**Figure 1:**
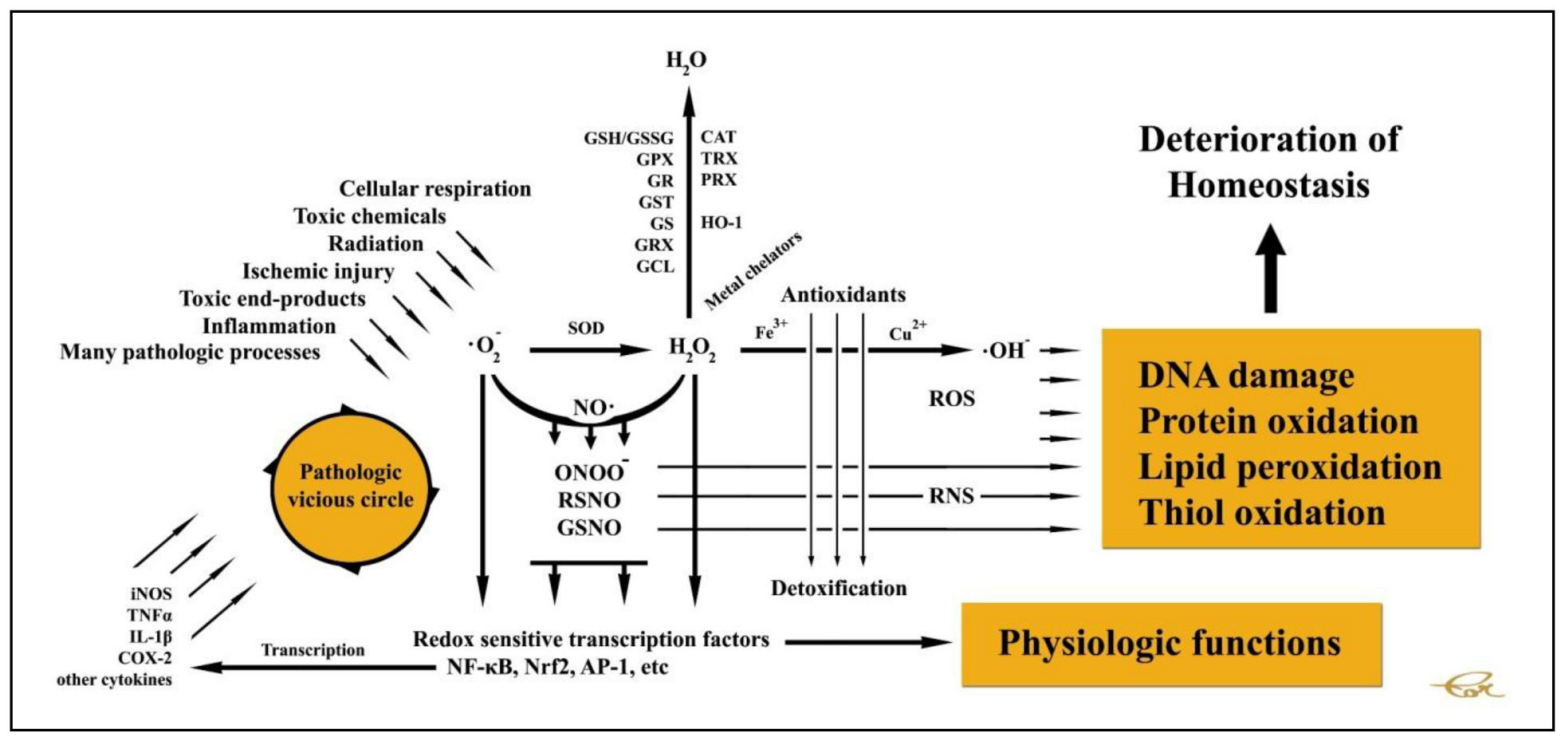
Major known oxidant/antioxidant pathways in living organisms. Many pathologic processes including inflammation, ischemia, irradiation, *etc.* as well as physiological functions such as cellular respiration can trigger or increase superoxide radical (O_2_^•−^) production. The antioxidant enzyme superoxide dismutase (SOD) facilitates the dismutation reaction of O_2_^•−^ to hydrogen peroxide (H_2_O_2_). H_2_O_2_ can be reduced to water (H_2_O) via different ways: the glutathione cycle in which the reduced form of glutathione (GSH) was oxidized to glutathione disulfide (GSSG) and then will again be reduced to GSH plays the major role; mainly glutathione peroxidase (GPX) glutathione reductase (GR) and glutathione-S-transferase (GST), but also glutathione synthethase (GS), glutaredoxin (GRX) and glutamate cysteine ligase (GCL) are involved in this system. Catalase (CAT), thioredoxin (TRX) and peroxiredoxin (PRX) are also fighting against H_2_O_2_ overproduction; heme oxygenase-1 (HO-1) and several metal chelators are other important members of the endogenous redox state regulatory systems. If free Fe^3+^ or Cu^2+^ are present around H_2_O_2_, another possible - but unwanted - pathway is the production of the hydroxyl radical (^•^OH), one of the most reactive species known, via the Fenton and following Haber-Weiss reactions; ^•^OH have the ability to oxidize almost all biomolecules. Another unwanted pathway is, in the presence of excessive amounts of nitric oxide (NO^•^) produced mainly by the inducible isotype of nitric oxide synthase (iNOS), the outcompeting of SOD for its substrate O_2_^•−^. In this case the reaction of NO^•^ with O_2_^•−^ will produce peroxynitrite (ONOO^−^), a highly reactive molecule; S-nitrothiols (RSNOs) such as S-nitrosoglutathione (GSNO) can be produced in following steps. On the other hand, the radical or non-radical reactive species can trigger the activation of ‘redox sensitive transcription factors’ such as nuclear factor kappa B (NF-κB) or activator protein-1 (AP-1) which can mediate both a lot of physiological functions or inflammatory responses via several cytokines. Particularly in chronic pathologies, the re-activation of reactive molecules can lead to a vicious circle. Please note that **‘antioxidants’** cover a large broad of molecules and they can act in much more steps of redox reactions than simply shown in the figure; *e.g.* inhibiting ROS generating enzymes, supporting the production of endogenous defense molecules, scavenging free radicals. [Other abbreviations: ROS, reactive oxygen species; RNS, reactive nitrogen species; TNFα, tumor necrosis factor alpha; IL-1β, interleukin 1-beta; COX-2, cyclooxygenase 2; Nrf2, Nuclear factor erythroid 2-related factor 2]
